# ComPara: A corpus linguistics in English of computation in architecture dataset

**DOI:** 10.1016/j.dib.2022.108169

**Published:** 2022-04-12

**Authors:** Anca-Simona Horvath

**Affiliations:** Research Laboratory for Art and Technology, Department of Communication and Psychology, Aalborg University, Rendsburggade 14, 6212, 9000 Aalborg, Aalborg, Denmark

**Keywords:** Computational architecture, Digital architecture, Parametric architecture, Digital construction, Natural language processing, Human-building interaction, Digitalization, Architectural theory

## Abstract

ComPara is a corpus linguistics dataset in English focused on computational architecture or architecture where technology functions as a driver for its conceptualization, design, and materialization. Sometimes computational architecture is also referred to as digital, parametric, algorithmic or generative architecture, and, as has been shown, each of these terms has different flavours [9]. Other corpus linguistics for architecture have been built containing texts written over a relatively limited time span and focusing on the language used in the profession in general [1,2]. The text which makes up ComPara is written between 2005 and 2019 and focuses on computational architecture. The corpus is built from two sources: the journal *Architectural Design*[3] and the *eVolo skyscraper competition*[4]. The former is one of the journals which has focused most on the theoretical discourse surrounding computation in architecture [5], while the latter is one of the most prestigious competitions focusing on ‘technological advancements in architecture’ [4].

The corpus includes the titles of Architectural Design's journal issues, titles of all articles and the keywords which are associated to the *Introduction* article in the journal's web page for each issue for the period between 2005 to 2019. From the eVolo Skyscraper competition, the titles of all winning projects and honorable mentions as well as all abstracts describing the projects between 2006 and 2019 were collected. This amounts to around 100.000 words. The purpose of building this dataset was to help gain a better understanding of the digitalization of architecture over 15 year time-span [8]. Further quantitative, qualitative or mixed method analysis can be carried out using the ComPara corpus by following specific topics or trends over time or by comparing the corpus to other sources.

## Specifications Table

**Table:** 

Subject	Engineering
Specific subject area	Architecture
Type of data	.txt files .csv Files .pdf files .svg files
How data were acquired	By scraping the online repositories of the journals Architectural Design and the eVolo skyscraper competition.
Data format	Raw Filtered Analyzed
Parameters for data collection	For the journal Architectural Design: the titles of each issue, the titles of all articles and the keywords associated to the Introduction (2005-2019) were scraped from the journal's webpage. For the eVolo Syscraper competition: the titles, abstracts, authors and countries of winning projects and honourable mentions were scraped from the eVolo website.
Description of data collection	The data collection took place during one week in September of 2019 using the web scraping tool Octoparse [6] installed on a Windows machine. The data is separated in the two journals, and by year.
Data source location	The data was collected at: Institution: Aalborg University City/Town/Region: Aalborg Country: Denmark
Data accessibility	Repository name: Mendeley Data identification number: [10.17632/7ktscvmxvg.5] Direct URL to data: [https://data.mendeley.com/datasets/7ktscvmxvg/5]
Related research article	Anca-Simona Horvath: *How we talk(ed) about it: Ways of Speaking About Computational Architecture,* International Journal of Architectural Computing, 2022, https://doi.org/10.1177/14780771211070006

## Value of the Data

• This corpus is an insight on the language used to talk about architecture in one journal and one competition which have been focusing on technological advancements in the field. Architectural Design presents theoretical insights, and the eVolo skyscraper competition shows the language used to describe conceptual projects that have received awards or honourable mentions.
• Architectural theorists and historians can use the data to re-read the recent history of the section of architecture which focuses on technological advancements. Those who want to submit conceptual projects to the eVolo skyscraper competition can use this corpus to get a better understanding of the themes which have been successful through the years.
• The data can be used to gain a better understanding of how computation is penetrating the field of architecture over a 15 year period.
• The dataset can be processed quantitatively (for topic modelling by natural language processing), qualitatively or through mixed method approaches.

## 1 Data Description

ComPara is a collection of article titles, keywords, titles and abstracts of conceptual projects collected from two sources: the journal Architectural Design and the eVolo Skyscraper Competition, each with its own folder and subfolders.

The **Architectural Design (2005-2019)** folder contains three subfolders: (1) one for the journal titles, (2) one with article titles and (3) one with the keywords associated to the Introduction article in the journal's web page, of each journal issue.

The subfolder *Architectural Design journal titles 2005-2019* contains 15 .txt files with the titles of each issue organized year by year. There are 90 issue titles (6 per year), with 527 words with 282 unique word forms.

The subfolder *Architectural Design article titles 2005-2019* contains 15 .txt files with the article titles organized year by year and 15 .svg files representing word clouds of the 500 most used words in the articles for each year. There are 1692 articles titles in total with 12,022 total words with 3,972 unique word forms (year by year this means: 2005 = 80 articles, 2006 = 107 articles, 2007 = 116 articles, 2008 = 145 articles, 2009 = 154 articles,2010 = 122 articles, 2011 = 100 articles, 2012 = 120 articles, 2013 = 121 articles, 2014 = 107 articles, 2015 = 116 articles, 2016 = 104 articles, 2017 = 103 articles, 2018 = 104 articles, 2019 = 93 articles).

The subfolder *Architectural Design Introduction keywords 2005-2019* has 15 .txt files organized by year which contain the keywords associated to the Introduction article of each journal issue. These keywords were collected from the *Information* section next to the *Introduction* article on the journal's webpage. It seems that they are generated automatically using a language processing algorithm, but details of the algorithm are inaccessible to external users. These make up for 13,014 words with 5,961 unique word forms.

The **eVolo Skyscraper Competition (2006-2019)** folder contains one .pdf file and three subfolders namely: (1) one which has the data for the winning projects, (2) one which has the data for the honourable mentions, and (3) a third with visualized data on the countries of origin of all projects.

The subfolder *eVolo skyscraper competition winning projects 2006-2019* contains 14 .csv files – one for each year. The .csv headlines are: title of project, .url, authors, country under which authors register their project and the abstract describing the project.

Similarly, the *eVolo skyscraper competition honourable mentions 2006-2019* contains 14 .csv files – one for each year with the headlines: title of project, .url, authors, country under which authors register their project and the abstract describing the project.

The subfolder *eVolo Skyscraper competition countries of winning projects and honourable mentions* contains two .pdf files which show coloured world maps created from the country data of the projects. The idea was to illustrate how geographies are represented in the competition. The first map shows only the countries of winning projects, while the second shows the countries of winning projects and honourable mentions.

Finally, the .pdf file called *Wordclouds of all titles of winning projects and honourable mentions - per year (2006-2019)* contains word cloud visualizations of the titles of winning projects and honourable mentions ordered year by year.

There are 39 winning projects and 285 honourable mentions, making up for a total of 324 projects. The eVolo titles subcorpus has 1,367 total words and 698 unique word forms. The eVolo abstracts contain 85,793 words and 9,458 unique word forms.

## 2 Experimental Design, Materials and Methods

The corpus was built with the help of the web scraping tool Octoparse [6] from the websites of the journal Architectural Design and eVolo and the data collection was done in several steps detaild below.

For the journal *Architectural Design*, there were two steps: (a) the titles of the issues and the titles of the articles in each issue were collected – all this for the period between 2005 and 2019. (b) In the second step, the keywords for the associated to the Introduction article in the journal's web page of each issue were collected for the period 2005-2019. The .url for each issue was created automatically using a script made with the visual programming language Grasshopper [7]. This meant simply changing the year, volume number and issue number at the end of the .url string. These .urls were introduced into Octoparse as batches for every year between 2005 and 2019. For each yearly batch, an Octoparse job was created. The target data to be extracted from the batches was selected manually in the browser. This target data was: The title of the issue and the titles of the articles in each issue. Each yearly batch was placed in separate .txt file. The word clouds from the .txt files were created using the Cirrus function from Voyant Tools. The Cirrus function deletes any punctuation and connection words, only shows the most common 500 words, and dimensions them by frequency.

For the *eVolo Skyscraper Competition*, the data was collected in two steps: (1) first, the winning projects, and (2) second, the honourable mentions. .urls were created manually for each years’ winners and each years’ honourable mentions between 2006 and 2019. The .urls were used to create yearly batches in Octoparse and then jobs were created for each batch. The target data to be extracted was selected manually in the browser and it was: the titles of the projects, the authors, the country, and the abstract describing the project. Each yearly batch resulted in two .csv files, one with winning projects and one with honourable mentions. The word clouds of the project titles were created, again, using the Cirrus function from Voyant Tools. Then, two maps were created using a Grasshopper script which coloured the interior of the polyline of countries on a world map according to how many times a country appeared in the list of (1) winning projects and (2) honourable mentions and winning projects in the .csv file described above. The dataset can be found online at [10].

## Ethics Statement

Hereby, I Anca-Simona Horvath consciously assure that for the manuscript *ComPara: A Corpus Linguistics in English of Computation in Architecture Dataset*, the following is fulfilled:

1) This material is the authors' own original work, which has not been previously published elsewhere.
2) The paper is not currently being considered for publication elsewhere.
3) The paper reflects the authors' own research and analysis in a truthful and complete manner.
4) The paper properly credits the meaningful contributions of co-authors and co-researchers.
5) The scraping of Architectural Design's repository is only done on data available to the general audience who do not have to be registered customers to see titles of journal issues and articles and keywords associated to the Introduction article of each issue. Additionally, scraping is done in accordance to Wiley's Text and Data Mining Agreement.
6) The eVolo skyscraper competition repository is licensed under a Creative Commons License permitting non-commercial sharing with attribution.

The violation of the Ethical Statement rules may result in severe consequences.

I agree with the above statements and declare that this submission follows the policies of Solid State Ionics as outlined in the Guide for Authors and in the Ethical Statement.

28.03.2022

Anca-Simona Horvath

## CRediT authorship contribution statement

**Anca-Simona Horvath:** Conceptualization, Methodology, Software, Data curation, Writing – original draft, Visualization, Investigation, Writing – review & editing.

## Declaration of Competing Interest

The authors declare that they have no known competing financial interests or personal relationships which have or could be perceived to have influenced the work reported in this article.

## Data Availability

ComPara: A Corpus Linguistics Dataset of Computation in Architecture (Reference data) (Mendeley Data).

## Appendix Supplementary materials

Image, application 1
