# Supplementary material for: ComPara: A corpus linguistics in English of computation in architecture dataset
Source: Data Brief. 2022 Apr 12;42:108169. doi: 10.1016/j.dib.2022.108169 (PMC9062263; doi:10.1016/j.dib.2022.108169)
Supplement: Supplementary file 1 [file mmc1.doc]

**Ethical Statement**

Hereby, I Anca-Simona Horvath consciously assure that for the manuscript *ComPara: A Corpus Linguistics in English of Computation in Architecture Dataset*, the following is fulfilled:

1) This material is the authors' own original work, which has not been previously published elsewhere.

2) The paper is not currently being considered for publication elsewhere.

3) The paper reflects the authors' own research and analysis in a truthful and complete manner.

4) The paper properly credits the meaningful contributions of co-authors and co-researchers.

5) The scraping of Architectural Design’s repository is only done on data available to the general audience who do not have to be registered customers to see titles of journal issues and articles and keywords associated to the Introduction article of each issue. Additionally, scraping is done in accordance to Wiley’s Text and Data Mining Agreement.

6) The eVolo skyscraper competition repository is licensed under a Creative Commons License permitting non-commercial sharing with attribution.

The violation of the Ethical Statement rules may result in severe consequences.

I agree with the above statements and declare that this submission follows the policies of Solid State Ionics as outlined in the Guide for Authors and in the Ethical Statement.

Date: 22.02.2021

Corresponding author’s signature:

Anca-Simona Horvath
